# BLAST-EXPLORER helps you building datasets for phylogenetic analysis

**DOI:** 10.1186/1471-2148-10-8

**Published:** 2010-01-12

**Authors:** Alexis Dereeper, Stephane Audic, Jean-Michel Claverie, Guillaume Blanc

**Affiliations:** 1 Information Génomique & Structurale - CNRS-UPR2589, Institut de Microbiologie de la Méditerranée - IFR 88, 163 Avenue de Luminy, 13009 Marseille, France; 2 Current Address: INRA - UMR 1097, DIA-PC "Diversité et adaptation des Plantes Cultivées", 2 Place P. Viala, 34060 Montpelllier - France

## Abstract

**Background:**

The right sampling of homologous sequences for phylogenetic or molecular evolution analyses is a crucial step, the quality of which can have a significant impact on the final interpretation of the study. There is no single way for constructing datasets suitable for phylogenetic analysis, because this task intimately depends on the scientific question we want to address, Moreover, database mining softwares such as BLAST which are routinely used for searching homologous sequences are not specifically optimized for this task.

**Results:**

To fill this gap, we designed BLAST-Explorer, an original and friendly web-based application that combines a BLAST search with a suite of tools that allows interactive, phylogenetic-oriented exploration of the BLAST results and flexible selection of homologous sequences among the BLAST hits. Once the selection of the BLAST hits is done using BLAST-Explorer, the corresponding sequence can be imported locally for external analysis or passed to the phylogenetic tree reconstruction pipelines available on the Phylogeny.fr platform.

**Conclusions:**

BLAST-Explorer provides a simple, intuitive and interactive graphical representation of the BLAST results and allows selection and retrieving of the BLAST hit sequences based a wide range of criterions. Although BLAST-Explorer primarily aims at helping the construction of sequence datasets for further phylogenetic study, it can also be used as a standard BLAST server with enriched output. BLAST-Explorer is available at http://www.phylogeny.fr

## Background

The reconstruction of phylogenetic trees from molecular sequences has become a routine task not only for specialists involved in molecular evolution or systematics but also for biologists working on their favourite gene/protein family or annotating new genome sequences. The growing interest for phylogenetic information has stimulated the emergence of new integrated, user-friendly software that produce robust trees using sophisticated methods while remaining accessible to non-specialists. Developers concentrated most of their effort on improving the speed, accuracy and versatility of the algorithms proposed for reconstructing phylogenetic trees from user-defined sets of homologous (ancestrally related) sequences. However, albeit choosing a good initial sequence dataset is crucial to the validity of subsequent phylogenetic analyses, this step has been largely overlooked in recent software developments.

There is no single way for constructing datasets suitable for phylogenetic analysis, because this task intimately depends on the scientific question we want to address. For example, biologists may be concerned by the taxonomic range of sequences, reduction of long-branch attraction effect, presence of paralogues, orthologues, pseudo-genes and/or multi-domain proteins, etc. Failure in the constitution of datasets can lead to draw incorrect conclusion from phylogenetic studies. Tools have been specifically designed to distinguish between orthologous and paralogous in genome/proteome datasets [1] and expressed sequence tag datasets [2,3], but they not always the most convenient for punctual analyses.

A frequent scenario in research is a biologist having a particular sequence of interest in hands that needs to find other sequences that are related to it in sequence databases to create a phylogenetic tree. The Basic Local Alignment Search Tool (BLAST) [4] is the most widely used set of programs for this purpose, primarily owing to its speed of execution. However, the information presented in the traditional BLAST output is not optimized for selecting and retrieving items for further phylogenetic study. For example the ranking of the identified matches in function of the alignment scores do not reflect accurately the evolutionary distances between the query and matching sequences (subject sequences). This is mainly because the BLAST scoring scheme has bias favouring hits that have smaller or no gaps (keep hits as single, long, high-scoring local alignments) over hits with long gaps (provoke the split of hits in multiple, short, low-scoring local alignments). Furthermore, BLAST results only show the levels of divergence between the query sequence and each of the individual matches but not between pairs of matching sequences. Yet this information is important when one wants to control sequence diversity in the phylogenetic dataset (e.g., avoiding over-sampling of sequences arising from the same taxon). Many features of individual alignments are useful for sampling homologous sequences (e.g., alignment coverage, level of similarity, etc) but their organization and accessibility across the BLAST result page do not facilitate their interpretation.

To fill this gap, we created BLAST-Explorer. This web-based resource combines a fast parallelized BLAST search with a suite of tools that allows an interactive exploration of the BLAST results and the easy selection of a suitable subset of homologous sequences. The traditional BLAST output is entirely reformatted to highlight phylogeny-relevant information and augmented with new features not provided by BLAST (taxonomy information, multiple alignments and similarity trees). BLAST hits can be selected either individually or in bulk using various criterions. Selected items can then be imported locally as fasta-formated sequences or passed to one of the phylogenetic tree reconstruction pipelines available on the Phylogeny.fr platform [5].

## Implementation

### Initial BLAST search

Blast searches are parallelized on an in-house 25-node Linux cluster (50 CPUs) and accept either proteins or nucleic acids as query. Searches can be done using the BLASTP (protein against protein database) or BLASTN (nucleotides against nucleotide database) algorithms. The TBLASTN (amino-acids against a 6-frames translated nucleotides database) and BLASTX (translated nucleotides against a protein database) algorithms are also proposed but in this case, subsequent analyses (i.e., similarity tree and multiple sequence alignment) are carried out at the protein level. The use of TBLASTX is not proposed because its output is not manageable in subsequent post-processing (e.g., alignments in overlapping non-coding reading frames). Protein and nucleotide sequence databases are updated at monthly interval. The parallelization of the Blast searches is done as follows: in a first step, each compute node aligns the query sequence against a distinct subdivision of the selected sequence database using the specified BLAST program. Then the resulting hit sequences on each node are gathered together in a smaller database which is searched again in a second BLAST run for formatting the final output. In each BLAST run, the effective size of the generic database (i.e., NR, NT, etc.) is specified using the -z flag to allow accurate calculation of the alignment E-value.

#### BLAST output post-processing

The Blast output is parsed to collect various features (scores, pairwise alignments, sequence annotations, sequence identification numbers, e-values). For each hit, taxonomic information (species and lineage) is retrieved from a weekly updated local copy of the NCBI Taxonomy database ftp://ftp.ncbi.nih.gov/pub/taxonomy/. The Blast output is entirely redesigned such that the information most relevant to phylogenetic analysis becomes easily accessible. Menu panels and images of the similarity tree and tiling diagrams (see below) are also included.

### Construction of the similarity tree

This tool provides a phylogenetic-oriented graphical display of the BLAST results. First, a pseudo multiple-sequence alignment (MSA) of the query and BLAST hit sequences is created by parsing the standard BLAST output: individual BLAST-aligned hit sequences are piled up by positioning each residue relative to its homologue in the query sequence (high scoring pairs [HSP] stacking). Multiple non-overlapping HSPs for a same hit are concatenated; regions of the hit sequence not aligned with the query sequence are substituted with gaps. When duplicated domains are present in the hit sequence, each repeat unit produces a HSP with the homologous region in the query sequence. In this case, only the repeat unit contained in the highest scoring pair is included in the MSA (i.e., repeated domains with lower alignment scores are not considered in the alignment). Although pseudo MSAs may be less accurate than MSAs created by conventional programs (i.e., ClustalW, Muscle, etc.), we chose this option because it is much faster for large datasets (up to 5000 hit sequences).

This pseudo MSA is passed to ClustalW, which produces a similarity (p-distance) tree using the "-tree" option. This tree is built with the neighbor-joining method, using either all sites of the alignment or gap-free sites only, depending on the user choice. A picture of the similarity tree is generated by the TreeDyn program, using the "reporting annotations" functions for color-coding. This image is incorporated into the new HTML Blast output together with a map of embedded Javascript actions allowing the mouse-click selection of hits.

### Implementation

The Blast Explorer web interface and scripts are implemented in CGI/Perl. The interactive web page is powered by the Javascript and AJAX technologies. The HTML pages are best viewed on a 19-inches (or larger) screen.

## Results and discussion

### Running BLAST-Explorer

The entry page of BLAST-EXPLORER is a simplified BLAST form that receive a single fasta-formated query sequence as input and allows (i) the selection of BLASTN, BLASTP, TBLASTN, or BLASTX [4] as an alignment algorithm, (ii) the selection of a sequence database (Genbank NT for nucleotides; Genbank Non Redundant Protein, Ensembl, PDB, RefSeq, Uniprot and Swissprot for proteins), (iii) the selection of a BLAST E-Value threshold and (iv) the option of filtering out low-complexity sequence segments. BLAST searches report a maximum of 5,000 hits.

### Small scale selection mode

**Figure 1:**
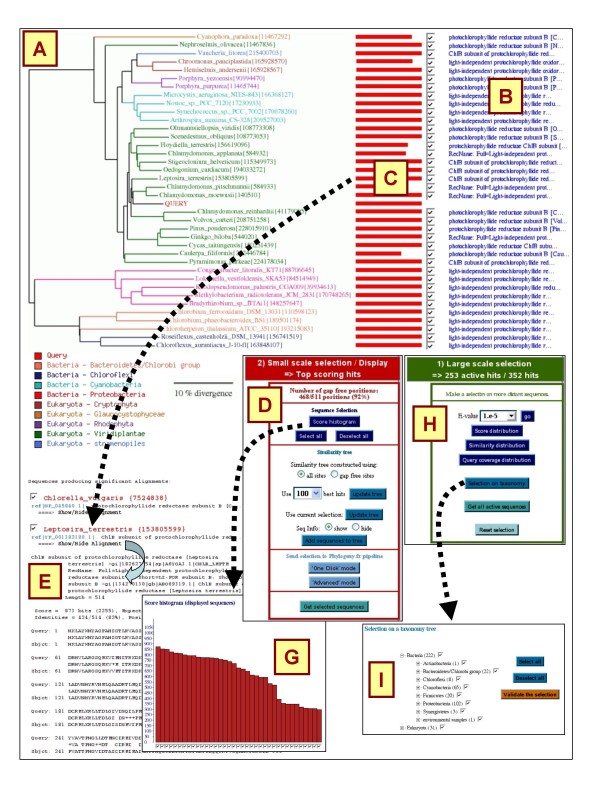
**BLAST-Explorer main interface**. BLAST-Explorer main interface showing the similarity tree (A), hit descriptions (B), a coverage diagram representing the alignment of the hit sequences on the query (C), the taxonomy color code (D), individual BLAST pairwise alignments (E), the small-scale (F) and large-scale (H) selection tool panels. The "Score histogram" tool (G) and "Selection on taxonomy" tool (I) are given as examples.

By default, the result page only shows the top-100 scoring BLAST hits, while the remaining hits are kept in memory and can be activated using the large-scale selection tools (next section). Small-scale selection tools only apply on the top-100 scoring BLAST hits. The central tool in this mode is the sequence similarity tree that provides an approximate picture of the phylogenetic relationships between the query and the top BLAST hits (Fig. 1A). BLAST hits are renamed according to the species name. The similarity tree is documented with meta-information including hit description (Fig. 1B), alignment coverage (Fig. 1C), taxonomy-based coloring (Fig. 1D). The tree image allows a navigation across the BLAST result page (clicking on an alignment coverage bar [Fig. 1C] leads to the corresponding pairwise alignment [Fig. 1E]), gives access to the database record (by clicking on the hit name), as well as to the selection of individual hits (check-boxes) or in bulk (by clicking on internal branches).

A dropdown menu (Fig. 1F) gives access to additional small-scale selection tools:

o The top-panel shows the number of gap-free sites in the BLAST-reconstructed multiple-alignment of selected sequences (see supplementary data). This number is dynamically updated when BLAST hits are added or removed from the selection.

o The "score histogram" tool shows the BLAST score values ranked in decreasing order. A score threshold can be applied by clicking on the histogram (e.g., Fig. 1G).

o Two "Update tree" options allow redrawing the similarity tree by setting the appropriate number of top-scoring BLAST hits or using a user-defined sequence selection. The tree is generated by combining ClustalW [6] and TreeDyn [7] using either all sites of the BLAST-reconstructed multiple-alignment or gap-free sites only (N.B., the initial tree is computed using all sites).

o The "Add sequences to tree" option allow incorporating up to five external sequences (supplied by users) into the current hit sequence selection. The similarity tree is then recalculated to show the phylogenetic position of the external sequences relative to the BLAST hit sequences.

At the end of the selection process, selected sequences can be imported in fasta format ("get selected sequence" button) or passed to one of the phylogenetic reconstruction pipelines available on the phylogeny.fr platform [5] ("One click mode" or "Advanced mode" buttons).

### Large-scale selection mode

In the large-scale selection mode, several tools allow the sampling of homologous sequences among the entire set of BLAST hits (including those that are not shown in the top-100 BLAST subset) using global criterions. They are grouped in a dedicated panel (Fig. 1H) and comprise:

o A pull-down menu that allows changing the e-value threshold on BLAST hits

o Buttons showing the distributions of the BLAST hits according to three BLAST alignment statistics (i.e., BLAST scores, percentage of similarity, and alignment coverage). Bulk selection among the BLAST hits can then be done by selecting intervals of the distribution histogram.

o The "selection on taxonomy" tool enabling the selection of BLAST hits according to their taxonomic rank (e.g., Fig. 1I). The taxonomic information is presented as a hierarchical graph allowing users to adjust the level of details that is relevant to their needs.

Following the application of the selection rules, the result page (i.e., the similarity tree and individual pairwise alignments) is updated to account for changes in the list of the top-100 best BLAST.

### Comparison with existing software

Several existing BLAST post-processors combine BLAST searches with automated phylogenetic analysis of the BLAST hits. However most of them do not pursue the same goal and therefore differ in the nature of the results. Also, the functionalities proposed to interact with the results vary greatly. Some of the applications allow filtering of the BLAST hits before phylogenetic reconstruction, others do not.

Phylogena is a standalone application for phylogenetic annotation of unknown sequences [8] and implements an automated intelligent filtering of BLAST hits before phylogenetic reconstruction. In contrast with BLAST-Explorer, the hit filtering method is optimized for sequence annotation and do not enable interactive and progressive refinement of the sequence dataset. Furthermore Phylogena does not allow retrieving the selected sequences for external analysis.

Phylogenie is also a standalone application for automated phylome generation and analysis [9]. Because the principal force of Phylogenie is to automatically produce a large number of phylogenetic analyses in batch, it does not allow interactive filtering of BLAST hits before phylogenetic reconstruction. Phylogenie is a command-line driven pipeline, requiring at least some familiarity with UNIX and command line tools.

Phyloblast [10] and the NCBI BLAST server [11] are two web services that have the most in common with BLAST-Explorer. They produce an enriched BLAST output and allow selection of hits using various criterions. The Phyloblast server is apparently no longer maintained. Phyloblast only allowed comparing a protein sequence against a protein database using BLASTP whereas BLAST-Explorer allows nucleotide/nucleotide, protein/protein and translated nucleotide/protein comparisons. Tools for selecting hits before phylogenetic reconstruction are less versatile than those proposed by BLAST-Explorer (selection based on species names and sequence description). The NCBI BLAST service also provides several tools for selecting and retrieving matching sequences from the BLAST output; a distance tree of the BLAST hits can also be calculated. Here again the hit selection tools are more limited than in BLAST-Explorer (simple check boxes beside sequence descriptions). Furthermore the image of the distance tree does not allow interactive selection of the BLAST hits. This makes selection on phylogenetic criterion less straightforward.

The principal strength of BLAST-Explorer is the flexibility of the sequence selection process and the richness of the information displayed on screen. However, BLAST-Explorer does not propose pre-defined automated methods of hit selection such as for example in Phylogena. Rather, BLAST hit selection is multi-dimensional and mainly human-driven though an interactive graphical interface in order to respond to a wide range of sequence selection strategies. Another feature that differentiates BLAST-Explorer from other software is that it is entirely web-based. Thus no installation on personal computer and no regular update of the sequence databases are required.

The BLAST-Explorer output includes a phylogenetic representation of the BLAST hits (i.e., the similarity tree) that aims at helping in the hit selection process. It is important to note that this tree is not optimized for phylogenetic accuracy. Rather, we opted for a fast tree reconstruction strategy that is however sufficiently robust for providing an approximate phylogenetic position of the BLAST hits. Thus we advise users to use external specialized software if they want to improve or confirm the accuracy of the phylogenetic tree.

Finally, it is important to note that in some phylogenetic aspect, the the importance is a correct distinction between orthologous and paralogous sequences

## Conclusions

BLAST-Explorer provides a simple, intuitive and interactive graphical representation of the BLAST results that can greatly help biologists in building their sequence datasets prior to phylogenetic studies.

## Availability and requirements

• **Project name: **BLAST-Explorer

• **Project home page: **http://www.phylogeny.fr (direct link: http://www.phylogeny.fr/version2_cgi/one_task.cgi?task_type=blast).

• **Operating system(s): **Platform independent

• **Programming language: **Perl/CGI, javascript

• **Other requirements: **best viewed on a 19-inches (or larger) screen

• **Any restrictions to use by non-academics: None**

## Authors' contributions

AD carried out most of the programming work and drafted the manuscript. SA participated in the programming. JMC participated in the design and coordination of the project and drafted the manuscript. GB conceived of the study, and participated in its design and coordination, participated in the programming and drafted the manuscript. All authors read and approved the final manuscript.
